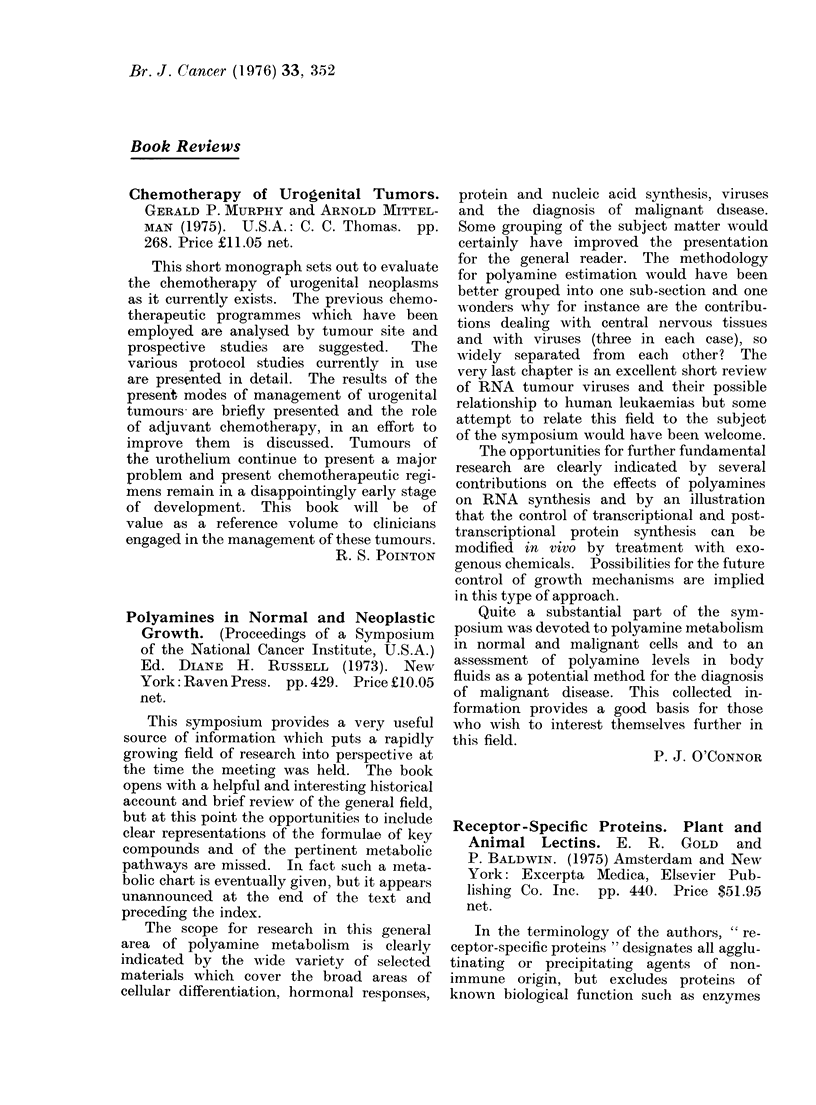# Chemotherapy of Urogenital Tumors

**Published:** 1976-03

**Authors:** R. S. Pointon


*Br. J. Cancer* (1976) **33**, 352

### *Book Reviews*

**Chemotherapy of Urogenital Tumors.** GERALD P. MURPHY and ARNOLD MITTELMAN (1975). U.S.A.: C. C. Thomas. pp. 268. Price £11.05 net.

This short monograph sets out to evaluate the chemotherapy of urogenital neoplasms as it currently exists. The previous chemotherapeutic programmes which have been employed are analysed by tumour site and prospective studies are suggested. The various protocol studies currently in use are presented in detail. The results of the present modes of management of urogenital tumours are briefly presented and the role of adjuvant chemotherapy, in an effort to improve them is discussed. Tumours of the urothelium continue to present a major problem and present chemotherapeutic regimens remain in a disappointingly early stage of development. This book will be of value as a reference volume to clinicians engaged in the management of these tumours.

R. S. POINTON